# Congenital Insensitivity to Pain: Novel *SCN9A* Missense and In-Frame Deletion Mutations

**DOI:** 10.1002/humu.21325

**Published:** 2010-09

**Authors:** James J Cox, Jony Sheynin, Zamir Shorer, Frank Reimann, Adeline K Nicholas, Lorena Zubovic, Marco Baralle, Elizabeth Wraige, Esther Manor, Jacov Levy, C Geoffery Woods, Ruti Parvari

**Affiliations:** Department of Medical Genetics, University of CambridgeUK; Department of Virology and Developmental Genetics, Faculty of Health Sciences, Ben Gurion University of the NegevIsrael; National Institute of Biotechnology in the Negev, Ben Gurion University of the NegevIsrael; Division of Pediatrics, Soroka Medical Center and Faculty of Health Sciences, Ben Gurion University of the NegevIsrael; Department of Clinical Biochemistry, University of CambridgeUK; International Centre for Genetic Engineering and BiotechnologyTrieste, Italy; Guy's and St Thomas' NHS Foundation TrustLondon, UK; Institute of Genetics, Soroka Medical Center and Faculty of Health SciencesBeer Sheva, Israel

**Keywords:** *SCN9A*, Sodium channel Na_v_1.7, congenital insensitivity to pain, channelopathy

## Abstract

*SCN9A* encodes the voltage-gated sodium channel Na_v_1.7, a protein highly expressed in pain-sensing neurons. Mutations in *SCN9A* cause three human pain disorders: bi-allelic loss of function mutations result in Channelopathy-associated Insensitivity to Pain (CIP), whereas activating mutations cause severe episodic pain in Paroxysmal Extreme Pain Disorder (PEPD) and Primary Erythermalgia (PE). To date, all mutations in *SCN9A* that cause a complete inability to experience pain are protein truncating and presumably lead to no protein being produced. Here, we describe the identification and functional characterization of two novel non-truncating mutations in families with CIP: a homozygously-inherited missense mutation found in a consanguineous Israeli Bedouin family (Na_v_1.7-R896Q) and a five amino acid in-frame deletion found in a sporadic compound heterozygote (Na_v_1.7-ΔR1370-L1374). Both of these mutations map to the pore region of the Na_v_1.7 sodium channel. Using transient transfection of PC12 cells we found a significant reduction in membrane localization of the mutant protein compared to the wild type. Furthermore, voltage clamp experiments of mutant-transfected HEK293 cells show a complete loss of function of the sodium channel, consistent with the absence of pain phenotype. In summary, this study has identified critical amino acids needed for the normal subcellular localization and function of Na_v_1.7. © 2010 Wiley-Liss, Inc.

## INTRODUCTION

Pain is one of the most pervasive symptoms in clinical medicine; it occurs in a multitude of clinical conditions and is encountered by clinicians in every subspecialty. Yet treatment of pain remains challenging despite many analgesic drugs and treatments with up to 50% of treated subjects receiving inadequate pain relief [Stewart et al., 2003]. Thus there exists a significant need to develop better therapies. In this paper we explore one method to achieve this goal: the analysis of gene mutations in humans with altered pain perception.

Over the past several years, elucidation of the genetic defects underlying three monogenic pain disorders has provided important insights about an unexpected component of human pain [Yang et al., 2004; Fertleman et al., 2006; Cox et al., 2006]. Channelopathy-associated Insensitivity to Pain (CIP) is a rare condition in which patients have no pain perception and anosmia, but are otherwise essentially normal (MIM# 243000). Recently, genetic studies in families demonstrating recessively inherited CIP have identified nonsense mutations that result in truncation of the voltage-gated sodium channel type IX α subunit *(SCN9A),* a 113.5-kb gene comprising 26 exons (MIM# 603415) [Cox et al., 2006; Goldberg et al., 2007; Ahmad et al., 2007; Nilsen et al., 2009]. The encoded sodium channel is composed of 1977 amino acids and is organized into 4 domains, each with 6 transmembrane segments [Klugbauer et al., 1995]. The *SCNA* family of sodium channels *(SCN1A-SCN11A)* evolved from an archetypal potassium channel by quadruplication, where four potassium subunits have to coalesce to form the functional potassium channel. *SCN9A* is predominantly expressed in the dorsal root ganglion (DRG) neurons and sympathetic ganglion neurons [Rush et al., 2006]. Functional studies, performed for only some mutations to date, have shown that CIP-associated mutations lead to a loss of function of Na_v_ 1.7 [Cox et al., 2006].

Heterozygous mutations that cause changes of amino acids in Na_v_ 1.7 result in very different pain phenotypes; Primary Erythermalgia (PE; MIM# 133020) and Paroxysmal Extreme Pain Disorder (PEPD; MIM# 167400) [Yang et al., 2004; Fertleman et al., 2006]. The primary symptoms associated with PE are severe chronic burning pain sensations in the hands and feet. The missense mutations causing PE are gain of function mutations that predominantly enhance the activation of Na_v_ 1.7 [Cummins et al., 2004]. PEPD is characterized by severe burning rectal, ocular, and submandibular pain sensations. Eight distinct Na_v_1.7 missense mutations were reported in different PEPD families that are different from those associated with erythermalgia [Fertleman et al., 2006]. The PEPD mutations that have been functionally characterized cause depolarizing shifts in voltage-dependence of steady-state inactivation and cause incomplete inactivation of Na_v_ 1.7, leading to prolonged persistent currents.

Recently, heterozygous missense mutations in *SCN9A* have been reported to cause febrile seizures, but without altered pain perception (MIM# 603415) [Singh et al, 2009]. These mutations are all in highly conserved amino acids and are postulated to alter the function of brain-expressed Na_v_1.7. Analysis of a knock-in mouse for one of the reported mutations (N641Y) showed homozygous mice to be significantly more susceptible to seizures [Singh et al, 2009], supporting the hypothesis that this mutation is disease-causing.

Thus prior to this study, missense mutations were only reported as leading to either excess or paroxysmal pain, or febrile seizures. Here we report for the first time missense mutations in *SCN9A* that cause CIP and demonstrate that the amino acid changes result in loss of function of Na_v_ 1.7.

## MATERIALS AND METHODS

### Patients and pedigrees

#### Bedouin Patients

**Figure 1:**
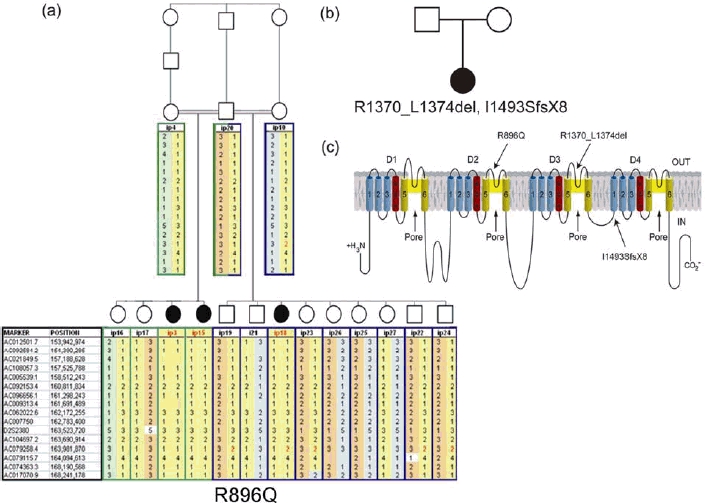
Novel mutations identified in *SCN9A*. **(a)** Pedigree and haplotype around the *SCN9A* gene of the Israeli Bedouin family with the Na_v_1.7-R896Q mutation; **(b)** Pedigree for British singleton carrying the two mutations Na_v_1.7-ΔR1370-L1374 and Na_v_1.7- I1493SfsX8; **(c)** Schematic representation of the Na_v_1.7 sodium channel and locations of the identified mutations. The plasma membrane is shown in grey; transmembrane segments are labelled 1-6; the extracellular linkers between transmembrane segments 5 and 6 form the channel pore. Note that the Na_v_1.7-R896Q and Na_v_1.7-ΔR1370-L1374 mutations both map to similar regions of the channel in domain (D) 2 and 3 respectively.

The patients belong to a consanguineous family, one father with 2 first degree cousin wives (Figure 1a). We studied three sisters of the same nuclear family. Two of the girls were the offspring of one wife and another patient was the daughter of the second wife to the same father. Their ages were 15, 5 and 3 years respectively. The pregnancy and delivery were normal. All presented with a history of indifference to pain in early childhood, e.g. pin pricks or falls, with recurrent trauma including skin burns, fracture of bones and amputation of toes which caused them little distress. All patients had old skin scars due to burns, frequent cuts and bruises. Deformities of the limbs due to osteomyelitis of the upper and lower limbs and old bone fractures were evident, including a fracture of the shoulder which was identified by local swelling in one patient and a tibial bone fracture which was noticed due to limping in another. Trauma of the oral region was frequent in all patients. The tips of the tongues were amputated due to recurrent biting. The patients extracted their teeth and suffered from recurrent bouts of mandibular osteomyelitis. On examination the children were able to feel fine touch and pin prick but were indifferent to painful stimuli caused by squeezing the Achilles tendon and finger tips. In addition, they showed a normal histamine flare response, sweated normally and had normal intelligence. Nerve conduction studies performed in 2 of the patients revealed normal motor conduction with borderline reduction in the medial plantar sensory action potential (SAP). Their corneal responses were reduced. The patients presented the clinical picture of CIP and had no features of other hereditary sensory neuropathies. Specifically, the phenotype differed from patients with Congenital Insensitivity to Pain with Anhydrosis (CIPA; MIM# 256800) in that all had normal cognitive development, lack of unexplained bouts of fever in infancy and early childhood, normal lacrimal product and sweat, and normal sympathetic skin responses. The study was approved by the Soroka Helsinki committee. See Supp. Methods for more information.

#### British patient

The first child of healthy, non-consanguineous parents was referred aged one year for investigation for insensitivity to pain. The only complication of pregnancy or delivery was maternal fever 5 days prior to delivery that was treated with antibiotics. Birth weight was 4.2 kg and there were no obstetric or neonatal problems. First concerns were raised at age 6 months when she would bite her fingers during teething, showing no distress with the consequent injury. She had failed to show any discomfort with earlier childhood vaccinations. Subsequent injuries, including abrasion of her feet when learning to walk with a push-along walker and ulceration of her tongue following biting, similarly resulted in no distress. Aged 4 years she sustained a fracture to a right metatarsal. The lack of pain response coupled with erythema initially resulted in misdiagnosis of cellulitis and there was a delay of weeks in radiographic assessment (Supp. Figure S1). Now aged 5 years she has been taught and reasoned with to no longer bite her fingers. She gets upset at the presence of blood but does not respond normally to painful events. She seems to have preservation of temperature perception. Autonomic function has been normal including normal sweat production, pupillary reflexes, temperature control, bladder and bowel function. Histamine flare test was normal. Cognitive development has been normal. On examination at one year there were scars from bites on her fingers, ulceration of her tongue and scarring from injury on her toes. Deep tendon reflexes and light touch sensation were normal. Fungiform papillae of the tongue were present. Corneal reflexes were absent. Motor nerve conduction studies were normal, peroneal sensory action potential (SAP) was absent and median and ulnar SAPs reduced. Sural nerve biopsy was normal histologically. Electron microscopy did reveal a few thinly myelinated axons surrounded by redundant Schwann cell basement membrane and although these could be interpreted to be in keeping with a demyelinating process, classical onion bulb formation was not present and there were no definitive abnormalities. A diagnosis of congenital insensitivity to pain was made and the normal development with absence of autonomic involvement led to consideration of *SCN9A* -associated pain insensitivity.

### Linkage analysis

For the Bedouin family polymorphic markers for the region were developed using Tandem Repeats Finder [Gelfand et al., 2007] and PCR primers designed by the use of the “Primer3” web site (http://frodo.wi.mit.edu/primer3/). PCR was performed on 50 ng DNA with the addition of 0.01 μCi α- [^32^P] dCTP to enable visualization of the PCR products which were separated on 6% polyacrylamide gels and visualized by a Phosphor–Imager [Parvari et al., 1998].

### Mutation screening

Genomic DNA was isolated from blood by standard methods. All coding exons and flanking splice sites of *SCN9A* were bi-directionally sequenced using either previously reported primers [Cox et al., 2006] or SCN9A ex 15f: GAGATATTGAAAATTGATGAAAATGA and SCN9A ex 15r: CAAAATTTCGTTCTCTTTCCTG. The c.2687G>A, c.4108_4122delCGATGGAAAAACCTG and c.4474delA mutations (NM_002977.3) were sought in 260 ethnically matched control chromosomes by amplicon restriction analysis with *BseDI* (Fermentas, Lithwania) (c.2687G>A) or bi-directional sequencing of genomic DNA. Nucleotide numbering reflects cDNA numbering with +1 corresponding to the A of the ATG translation initiation codon in the reference sequence, according to journal guidelines (http://www.hgvs.org/mutnomen).

### Verification of splicing

#### Direct PCR amplification of reverse transcribed RNA from patient's lymphoblastoid cells

RNA was extracted from lymphoblastoid cells of patient ip15 using the EZ-RNA II kit of Biological Industries (Israel) according to manufacturer instructions. cDNA was synthesized from 5 microgram RNA by the Reverse -iT 1^st^ strand synthesis kit of ABgene (Surrey, U.K.) with a decamer random primer. The PCR product confirming correct splicing was amplified using primers SCN9A-2310f: CCACCCAATGACTGAGGAAT and SCN9A-2977r: GGTTGTTTGCATCAGGGTCT, PCR conditions were: 45 cycles of 95° 1′, 57° 1′, 72° 1′ and elongation of 5′ at 72°. The PCR product was directly sequenced.

#### Minigene splicing assay

A DNA fragment of 1037 bp containing the wild-type coding exon 15 along with 321 bp of upstream and 360 bp of downstream intronic flanking regions was amplified from genomic DNA, sequenced and cloned into the *NdeI* site of the PTB minigene construct. The c.2687G>A mutation was created by PCR mutagenesis. Wild-type and mutant minigenes were transfected into HeLa cells using lipofectamine (Invitrogen). RNA was analysed by RT-PCR using primers specific for the minigene ALFA and BRA, followed by sequencing of the PCR products.

### Construction of expression plasmids

The wild-type *SCN9A* 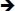 polio IRES 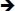 *DsRed2* construct (FLRED) and the *SCN1B* 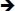 encephalomyocarditis virus (ECMV) IRES 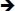 *SCN2B* 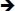 polio IRES 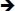 *EGFP* construct (JC5) were generated as previously described [Cox et al., 2006]. These bear either SCN9A and DsRed2 (FLRED) or SCN1B, SCN2B and EGFP (JC5) on the same vector expressed from the same promoter and the inclusion of *DsRed2* and *EGFP* in these constructs helped in the identification of positively co-transfected cells when patch clamping. The Na_v_1.7-R896Q and Na_v_1.7 -ΔR1370-L1374 mutations were introduced into FLREDN using the QuikChange XL Site-Directed Mutagenesis Kit (Stratagene) or by recombinant PCR respectively. The FLAG (DYKDDDDK) epitope was introduced into the above WT and mutant constructs into the domain I extracellular S1-S2 linker in frame between Pro 149 and Asp 150, using recombinant PCR techniques. For the immunocytochemistry experiments, the polio IRES-DsRED2 fragment was removed from the WT and mutant constructs so that only the Na_v_1.7 protein was expressed. All constructs were sequenced to verify the desired mutation and epitope and to ensure the lack of other introduced variations.

### Immunocytochemistry

Rat pheochromocytoma cells (PC12) cultured in DMEM supplemented with 10% horse serum and 5% FCS, were transiently transfected with the WT or either of the 2 mutant constructs using lipofectamine 2000. The JC5 construct was not co-transfected as endogenous beta subunits are present within PC12 cells. One day after transfection, the cells were fixed in ice-cold methanol for 5 mins and then permeabilized using acetone. Fixed cells were blocked with 3% BSA for 40 mins. Staining was carried out using the following antibodies: mouse monoclonal anti-Na_v_1.7 (1:250; Neuromab), mouse monoclonal anti-FLAG M2 (1:1000; Sigma), and rabbit polyclonal anti-Pan Cadherin (1:100; Sigma); all detected with appropriate Alexa Fluor secondary antibodies (1:250; Invitrogen). Slides were mounted using Prolong Gold antifade reagent with DAPI (Invitrogen) and examined using a Zeiss LSM510 META confocal laser-scanning inverted microscope (Carl Zeiss) equipped with an argon-krypton laser beam. At least 100 consecutive transfected cells per x3 coverslips were assessed for plasma membrane localization for each construct. To prevent bias the quantifying person was blinded against the nature of the analysed construct. Data was statistically analyzed using a Fisher's two-tailed exact test.

### Electrophysiology

HEK293A cells (QBiogene), cultured in DMEM supplemented with 5% FCS, were transiently transfected with plasmids expressing either WT or mutant Na_v_1.7 plus DsRed2 and/or SCN1B plus SCN2B plus EGFP using lipofectamine 2000. Whole-cell voltage clamp protocols were performed 2-3 days after transfection as previously described [Cox et al., 2006]. The bath solution contained (in mM): 3 KCl, 140 NaCl, 2 CaCl_2_, 1 MgCl_2_, 10 HEPES, 1 glucose (pH7.4 with NaOH) and the patch pipette solution contained (in mM): 107 CsF, 10 NaCl, 1 CaCl_2_, 2 MgCl_2_, 10 HEPES, 10 TEACl, 10 EGTA (pH 7.2 with CsOH).

## RESULTS

### Novel mutations identified in SCN9A

#### Mapping and DNA sequence analysis in an Israeli Bedouin family

Since the patients belong to the population of Bedouins in the south of Israel where congenital insensitivity to pain with anhydrosis (CIPA) is prevalent we firstly excluded linkage to the *TrkA* gene, assuming homozygosity in which the two alleles are identical by descent, by the use of microsatellite markers adjacent to the gene (data not shown). We next analyzed whether the patients presented homozygosity near the *SCN9A* gene, which was reported to be mutated in CIP patients [Cox et al., 2006; Goldberg et al., 2007; Ahmad et al., 2007; Nilsen et al., 2009]. Linkage to chromosome 2 (q23.3-q24.3), position 153,102,193–168,960,515 (NCBI Build 36.1 reference sequence) was confirmed by analysis of all family members for microsatellite markers in the region (Figure 1a). We searched for mutations in each of the coding exons and their intronic borders of this gene using DNA of patient ip3 and identified a single homozygous base substitution in coding exon 15 c.2687G>A resulting in amino acid change R896Q (reviewed but not shown). Arginine at position 896 is highly conserved, both evolutionary (Supp. Figure S2a) and in all sodium channels of the family (Supp. Figure S2b). This DNA change is not present in SNP or genomic databases. To exclude the possibility that the change is a population polymorphism, we determined its prevalence in healthy Bedouins of the same geographic region. The mutation eliminates a restriction site for the enzyme *BseDI* (Supp. Figure S3a). We analyzed 130 healthy Bedouins, using the elimination of a *BseDI* restriction site by the mutation, and found it was not present in any control individual (Supp. Figure S3b). However, since all 13 presently known mutations causing CIP truncate the protein, we verified that the substitution does not cause aberrant splicing. Firstly, bioinformatics analysis of the c.2687G>A coding exon 15 mutation using a battery of splice site/enhancer prediction algorithms (http://www.fruitfly.org/seq_tools/splice.html; http://rulai.cshl.edu/cgi-bin/tools/ESE3/esefinder.cgi?process=home) showed that splicing control was unlikely to be disrupted by the mutation. Secondly, RNA was extracted from lymphoblastoid cells of patient ip15, and an RT-PCR product was produced with primers fitting sequences in exons 14 and 16. The sequence of this product demonstrated that splicing was not altered (Supp. Figure S4a). Thirdly, we assessed the effect of the mutation on the control of splicing of exon 15 by a minigene assay. This showed that there was no difference between the wild-type and mutant minigenes with both producing mRNA with normal inclusion of coding exon 15 (Supp. Figure S4b).

#### DNA sequence analysis in the British patient

Sequence analysis of *SCN9A* in the British proband revealed that she was a compound heterozygote for a *de novo* five amino acid in-frame deletion (Na_v_1.7 -ΔR1370-L1374) and a truncating mutation (Na_v_1. 7-I1493SfsX8) (Figure 1b). Neither mutation is present in SNP or genomic databases or is predicted to disrupt splicing signals. Furthermore, these mutations were shown to be absent from 130 healthy Caucasian control individuals.

### Reduced cell surface expression of mutant channels

All channelopathy-associated insensitivity to pain mutations reported to date cause premature truncation of the Na_v_1.7 sodium channel [Cox et al., 2006; Goldberg et al., 2007; Ahmad et al., 2007; Nilsen et al., 2009]. Similarly, one of the mutant alleles in the compound heterozygote (Na_v_ 1.7-I1493 SfsX8) leads to truncation of the protein prior to domain 4 (Figure 1c), which would be predicted to cause a complete loss of function. However, the British five amino acid in-frame deletion (Na_v_1.7-ΔR1370-L1374) and the Bedouin missense mutation (Na_v_1.7-R896Q) are not expected to cause truncation but instead change the amino acid sequence in the S5-S6 extracellular loop of domains III and II of Na_v_1.7 respectively (Figure 1c). These loops form part of the sodium channel pore and both mutations are located just prior to the ion selectivity filter domain [Marban et al., 1998].

To study the effect of these mutations on the cellular localization of Na_v_1.7, FLAG-tagged *SCN9A* constructs were generated. The FLAG tag was inserted in the first extracellular loop in domain I of Na_v_1.7, similar to previous FLAG-tagging experiments performed for Na_v_1.5 [Makita et al., 2008; Liu et al., 2005]. Comparison of non-FLAG tagged and FLAG-tagged WT channels by patch clamping in HEK293 cells showed that epitope tagging did not affect the current densities or the gating properties of the sodium channel (Supp. Figure S5). Unfortunately, positively transfected cells could not be detected by immunostaining in a range of fixatives using a reliable anti-FLAG monoclonal antibody (data not shown). In HEK293 cells, we were also unable to see a convincing plasma membrane stain using a monoclonal anti-Na_v_1.7 antibody in cells transfected with the WT construct (data not shown).

**Figure 2:**
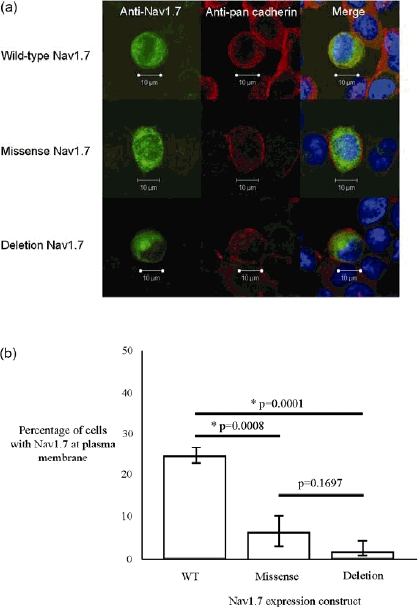
**(a)** Immuno staining of PC12 cells expressing the Na_v_1.7 sodium channel. From left to right the staining is as follows: anti-Na_v_1.7, anti-pan cadherin, merged image (with DAPI in blue). The WT Na_v_1.7 shows a faint but distinct 'rim' which colocalizes with the plasma membrane marker pan cadherin. Both the missense (Na_v_1.7-R896Q) and the in-frame deletion (Nav 1.7-ΔR1370-L 1374) typically do not show this rim effect. For each transfection experiment a representative cell is shown, with the Z slice chosen as the one with the most plasma membrane staining according to the anti-pan cadherin marker. Untransfected cells showed some golgi-localized staining, but never staining at the plasma membrane. **(b)** For each Na_v_1.7 construct, at least 300 cells were assessed for plasma membrane staining of Na_v_1.7. There were significantly more transfected cells with Na_v_1.7 staining at the plasma membrane for the WT transfections compared to each of the mutants (* indicates statistically significant result).

We therefore switched to analysis of the FLAG-tagged *SCN9A* constructs in undifferentiated PC12 cells. Using the monoclonal anti-Na_v_1.7 antibody we could see an obvious intracellular staining in cells transfected with the wild-type construct (Figure 2a and Supp. Figure S6). Furthermore, in a proportion of cells we could see a faint but distinct ‘rim’ of staining which co-localized with the plasma membrane marker pan cadherin (Figure 2a). In contrast, cells transfected with the mutant channels Na_v_1.7-ΔR1370-L1374 and Na_v_1.7-R896Q typically showed no plasma membrane staining for Na_v_1.7, although a similar number of cells as in the wild-type transfected cells showed strong intracellular staining. This suggests that the mutations caused abnormal trafficking of the channels to the plasma membrane compared to WT (Figure 2a and Supp. Figure S6). However, this phenotype was not the same in every cell as a minority of mutant-transfected cells appeared to show some Na_v_1.7 staining at the plasma membrane. To quantify this result we counted at least 300 transfected cells over 3 coverslips for each construct (Figure 2b). This showed that there were significantly more WT-transfected cells with Na_v_1.7 plasma membrane staining than the number of mutant-transfected cells with Na_v_1.7 plasma membrane staining. This data suggests that both the missense and in-frame deletion pore mutations hamper the surface expression of Na_v_1.7, and may explain the reason for the loss of pain phenotype seen in these patients.

### Mutations cause a complete loss of function of Na_v_1.7

**Figure 3:**
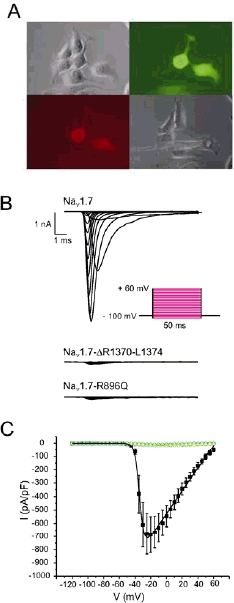
Electrophysiological characterisation of HEK293 cells transiently transfected with wild-type and mutant *SCN9A*. **(A)** Phase contrast, EGFP and DsRed2 fluorescence of HEK293 cells transiently co-transfected with plasmids expressing *SCN9A+DsRed2* and *SCN1B+SCN2B+EGFP* under a CMV promoter. Note that in the phase contrast with the patch pipette one of the fluorescently positive cells is missing as it was used in the previous patch experiment. **(B)** Current responses to 50 ms voltage steps in 5 mV increments between −110 and +60 mV from a holding potential of −100 mV, in a whole cell voltage clamp recording applied at ∼0.5 Hz for cells co-expressing either WT or mutant Na_v_1.7 (as indicated) with the β-subunits, identified by their fluorescence as shown in (A). The inset shows the voltage pulse protocol. **(C)** Current-voltage relationship of peak currents as shown in (B) normalised for cell size (pA/pF). ▪ Na_v_1.7+Na_v_β1+Na_v_β2 (n=5), 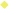 Na_v_1.7-ΔR1370-L1374+Na_v_β1+Na_v_β2 (n=5), 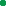 Na_v_1.7-R896Q+NaVβ1+Na_v_β2 (n=7), 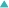 Na_v_β1+Na_v_β2 only (n=5). WT data was fitted with a Boltzmann equation y = (A2+(A1-A2)/(1+exp((V_0_._5_-x)/k)(x-V_rev_), where V_0_._5_ = −33 mV, k= 2 mV V_rev_= 65 mV.

Given that plasma membrane staining was occasionally seen when the mutant channels were overexpressed, we decided to investigate how the mutations affected the biophysical properties of the sodium channel. To do this, plasmids bearing either WT *SCN9A* or the mutant sequences were co-expressed in HEK293A cells with the auxiliary sodium channel β_1_ and β_2_ subunits (encoded by *SCN1B* and *SCN2B*). Whole cell voltage clamp recordings from cells co-expressing wild-type Na_v_1.7 with the β_1_β_2_ subunits, revealed a voltage-gated Na^+^ current with a peak amplitude of −685 ± 134 pA/pF at −20 mV (n=5), compared with a background current of −13 ± 2 pA/pF (n=5) in cells transfected with the β_1_ β_2_ construct alone (p=0.001 for wild-type Na_v_ 1.7 vs control) (Figure 3). The voltage dependence of activation of Na_v_1.7 could be described by a Boltzmann function, with half maximum activation (V_0.5_) of −33± 2 mV, k = 2 ± 1 mV and a reversal potential (V_rev_) of +65 ± 4 mV (n=5). Voltage dependent inactivation could also be described by a Boltzmann function with half maximal inactivation at −74 ± 2 mV and k = 4.3 ± 0.6 mV (n=5) (Supp. Figure S5). These properties are similar to those described previously for Na_v_1.7 current [Klugbauer et al., 1995; Cummins et al, 2004; Herzog et al., 2003]. In contrast, cells co-transfected with either of the mutated Na_v_1.7 subunits plus β_1_β_1_, exhibited currents that were not significantly different from those recorded from control cells (Figure 3). The mean peak currents at −20 mV were: −11±3 pA/pF (n=7, p>0.6 vs control) and −13 ± 5 pA/pF (n=5, p>0.9 vs control) for Na_v_1.7-R896Q and Na_v_1.7-ΔR1370-L1374 respectively. Hence, the two mutations completely abolish the function of the voltage-gated sodium channel, which is consistent with the complete insensitivity to pain phenotype seen in affected individuals from these two families.

## DISCUSSION

The ability to treat pain will hopefully improve upon our understanding of its molecular basis. The Na_v_1.7 voltage-gated sodium channel is a major and non-redundant part of pain perception and the identification of mutations that change its function holds the potential to contribute to pain relief. In particular, identification of the structural regions that are critical for the normal function of Na_v_1.7 is important as these regions could be targeted for the development of novel analgesics. However, all the CIP mutations reported to date have been truncating mutations, thus failing to reveal specific amino acids that are critical for normal function of Na_v_1.7. Here we present the first CIP missense and in-frame deletions in *SCN9A.* These null mutations highlight specific functionally significant amino acids, both being in the same functional domain of the Na_v_1.7 protein.

Previously reported missense mutations in Na_v_1.7 have been associated with PE, PEPD or febrile seizures and are mostly located to cytoplasmic linkers of the sodium channel, with some PE causing mutations in transmembrane domains. While it is not clear how and why these different gain-of-function mutations result in distinct disorders, it seems that PE causing mutations typically cause a hyperpolarizing shift in the voltage dependence of channel activation and a delay in deactivation of active channels upon return to non-activating membrane voltages. Both would increase the Na_v_1.7 currents at small deviations from the resting potential and the likelihood of action potential initiation, presumably sensitizing the neurons expressing these channels to small depolarising stimuli. PEPD causing mutations by contrast typically result in depolarizing shifts in the voltage dependence of fast inactivation, which is also seen in some PE-causing mutations, but importantly also rendering this process incomplete, thus resulting in persistent inward currents again leaving nociceptive neurons expressing these channels hyperexcitable [Drenth and Waxman, 2007]. More recently a mutation (Nav1.7-A1632E) which results in hyperpolarising shift in the voltage dependence of activation concomitantly with partial fast inactivation has been described in a patient with symptoms of both PE and PEPD [Estacion et al., 2008].

The missense mutation and in-frame deletion described here both map to the central sodium influx pore region of Na_v_1.7. The pore is formed by asymmetric loops (P segments) contributed by each of the four domains of the protein. The P segments are accessible only from the extracellular surface, with each undergoing a hairpin turn in the permeation pathway such that amino acids on both sides of the putative selectivity filter line the outer mouth of the pore. Evolutionary conservation of the pore helix motif from bacterial potassium channels to mammalian sodium channels identifies this structure as a critical feature in the architecture of ion selective pores [Yamagishi et al., 2001]. The mutations we describe both cause a significant reduction in the number of cells with plasma membrane staining of Na_v_1.7 by immunocytochemistry in transfected PC12 cells. This is similar to what happens for specific mutations in *SCN5A,* where the mutant channel becomes retained in the endoplasmic reticulum (ER) [Liu et al., 2005; Baroudi et al., 2001]. It is therefore conceivable that the *SCN9A* mutations described here also cause misfolding of the channel which leads to ER retention and hence defective cell surface localization of Na_v_1.7. However, as a minority of cells overexpressing the mutant protein appeared to show some plasma membrane staining, it seems reasonable to hypothesize that even if some mutant protein can make it to the membrane, insufficient current densities are reached, possibly due to malfolding of the channel pore. Whichever is the principal mutational mechanism, the result is a non-functional sodium channel causing congenital insensitivity to pain in the patients.

In summary, we have identified two novel non-truncating mutations in *SCN9A* which further expands the spectrum of mutations seen in Channelopathy-associated Insensitivity to Pain. The location of the mutations within the channel pore highlights the importance of this structure to channel function and shows how the highly regulated folding of this region can be disrupted by the alteration of a single amino acid. For analgesic design it suggests further possibilities: design of a moiety that can bind to the Na_v_1.7 sodium pore; and discovery, and then inhibition, of the chaperone protein that correctly folds Na_v_1.7 prior to its trafficking to the membrane.

## Supplemental material

**Supp. Figure S1.** Images of the painless compacted fracture suffered by the British patient.

**Supp. Figure S2.** Conservation of arginine at position 896. **(a)** Evolutionary conservation; **(b)** conservation across Na channel family members.

**Supp. Figure S3.** Analysis of the prevalence of the mutation in the patients' population. **(a)** Schematic demonstration of the analysis, a 693bp PCR product is cut by the restriction enzyme *BseDI*; **(b)** PCR products after BseDI restriction separated on a 2% agarose gel. DNA molecular weight marker is shown in the far right lane.

**Supp. Figure S4.** The c.2687G>A mutation does not alter splicing. **(a)** Partial sequence chromatogram of the RT-PCR spanning exons 14 to 16. The mutation is shown by an arrow. The nucleotides at the normal splicing sites at both sides of exon 15 are boxed. **(b)** Minigene assay to investigate whether the c.2687G>A mutation disrupts splicing control of *SCN9A*. The reporter gene is composed of α-globin (black boxes) and fibronectin exons (shaded boxes). WT or mutant coding exon 15 along with upstream and downstream intronic sequence were cloned into the *NdeI* site of the PTB minigene construct followed by transfection into HeLa cells. RT-PCR analysis shows that in the wild-type context (Lane 1) a PCR product of 594 bp was amplified which represented normal inclusion of exon 15 in the mature transcript. The minigene carrying the mutation behaved in an identical manner (Lane 2).

**Supp. Figure S5.** Voltage dependent activation and inactivation of wild-type and FLAG-tagged Na_V_1.7 channels. **(A)** (*left*) Current responses to 50 ms voltage steps in 5 mV increments between −110 and +60 mV from a holding potential of −100 mV, in a whole cell voltage clamp recording applied at 0.5 Hz for a cell coexpressing FLAG-tagged Na_V_1.7 with the α-subunits. The inset shows the voltage pulse protocol. (*right*) Current-voltage relationship of peak currents as shown on the left normalised for cell size (pA/pF). ▪ Na_V_1.7+Na_V_β1+Na_V_β2 (n=5),  FLAG-tagged Na_V_1.7+Na_V_β1+Na_V_β2 (n=5). The data was fitted with a Boltzmann equation y = (A2+(A1-A2)/(1+exp((V_0.5_-x)/k)(x-V_rev_). There was no significant difference between currents from wild-type and FLAG-tagged Na_V_1.7-expressing cells, with V_0.5_ = −33±1 mV and −33±2 mV; k=2±1 mV and 3±1 mV; and V_rev_ = 65±4 mV and 67±4 mV respectively. The peak current for FLAG-tagged Na_V_1.7 at −20 mV was 591±115 pA/pF which was not significantly different from wild-type expressing cells. **(B)** (*left*) Current responses of a FLAG-tagged Na_V_1.7+Na_V_β1+Na_V_β2 expressing cell in whole cell voltage clamp to a 50 ms test pulse to a potential of −10 mV applied immediately after 500 ms conditioning voltage steps to potentials from −120 to 0 mV (at 5 mV increments). The inset shows the voltage pulse protocol applied at 0.5 Hz. (*right*) Voltage dependence of the steady-state inactivation assessed as on the left for ▪ NaV1.7+Na_V_β1+Na_V_β2 (n=5),  FLAG-tagged Na_V_1.7+Na_V_β1+Na_V_β2 (n=5). The data was fitted with a Boltzmann equation y = (A1-A2)/(1+exp(x-V_0.5_)/k)) + A2. There was no significant difference between currents from wild-type and FLAG-tagged Na_V_1.7-expressing cells, with V_0.5_ = −74±5 mV and 76±1 mV and k= 4±1 mV and 6±1 mV respectively.

**Supp. Figure S6.** Immunostaining of PC12 cells expressing the Na_v_1.7 sodium channel. From left to right the staining is as follows: anti-Na_v_1.7, anti-pan cadherin, merged image (with DAPI in blue). These low magnification images illustrate that there was equal transfection efficiency for each of the constructs.

### SUPP. METHODS

**Additional Bedouin family clinical data**

The patients did not have bouts of unexplained fever (but had fever during infections), they had no dysphagia or vomiting. They had tears during emotional upset and had normal sweating. All had good bladder control (the youngest patient has nocturnal enuresis). One of the patients had a corneal abscess treated with parenteral antibiotics followed by tarsoraphy. According to the parents the children were indifferent to some flavours such as to an excess of salty food. (They appreciate food by vision). The patients were considered by their parents to have a well developed cognitive state, the elder one attended school. On examination the children were co-operative with well developed verbal skills and normal voices. They were oriented to place, time and to people. The ocular movements were normal, the pupils were round, equal and reacting to direct and consensual light with the presence but reduced corneal reflex and with lacrimation. The fundoscopic examinations were normal. Facial movements were normal. The tongues were normal with no fasciculation and with the presence of fungiform papillae. The uvulas were at the midline with intact gag reflex and swallowing. The tone was mildly reduced with normal bulk of muscles. The strength at both upper and lower limbs was 5/5. Deep tendon reflexes and plantar responses were normal. Cerebellar functions were normal. The gait and stance were normal. A mild laxity of joints was noticed. The motor conduction studies of the right peroneal and left tibial nerves showed normal distal latencies amplitudes and velocities. All patients had sweat in the armpits and on the palmar regions. There was no superficial peripheral nerve enlargement on palpation.

The patients were tested by the following methods: Motor and sensory nerve conduction studies as well as the sympathetic skin responses were performed on a Keypoint machine (Medtronic, Copenhagen, Denmark) using standard methods.

The motor nerve studies included the peroneal, tibial and median nerves.

The sensory studies included the medial planar and median nerves.

The sympathetic skin responses were recorded from the palms of hands.

Heart rate variability was evaluated during resting and deep breath.

Schirmer test was performed by applying film in the tarsal plate and measuring the length of wet film.

Sweat test was performed by applying a scopolamine patch.

Histamine test was performed by pin-pricking with histamine and measuring the flare responses.

The results of the tests: The motor conduction studies of tibial, peroneal and median nerves showed normal distal latencies amplitudes and velocities. The sensory studies of the medial plantar nerves showed normal latencies amplitudes and velocities. Sympathetic skin responses of the median nerves recorded from the palm were normal in all patients to electrical stimulation of the palm.

Sweat test, Schirmer test and Histamine test were all normal.
