# Supplementary figures and images for: Congenital Insensitivity to Pain: Novel SCN9A Missense and In-Frame Deletion Mutations
Source: Hum Mutat. 2010 Sep;31(9):1670–86. doi: 10.1002/humu.21325 (PMC2966863; doi:10.1002/humu.21325)

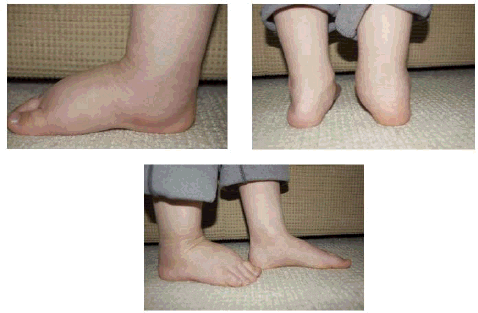

Supplement: Supplementary file 1 [file humu0031-1670-SD1.gif]

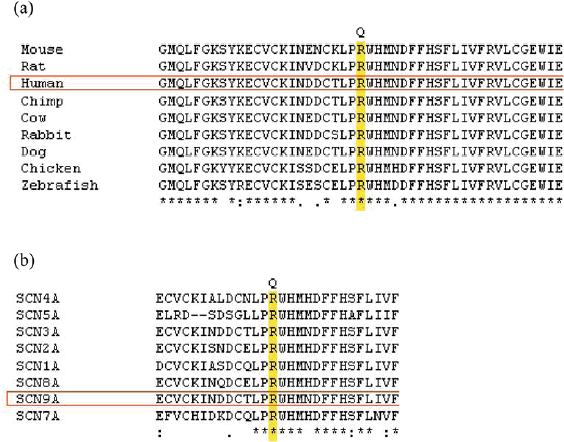

Supplement: Supplementary file 2 [file humu0031-1670-SD2.gif]

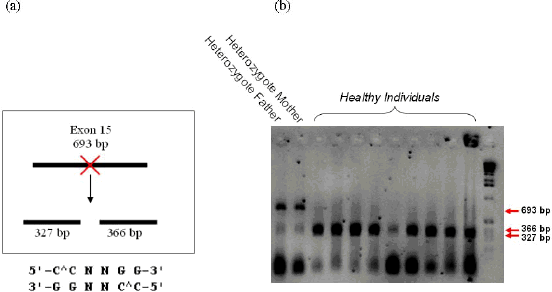

Supplement: Supplementary file 3 [file humu0031-1670-SD3.gif]

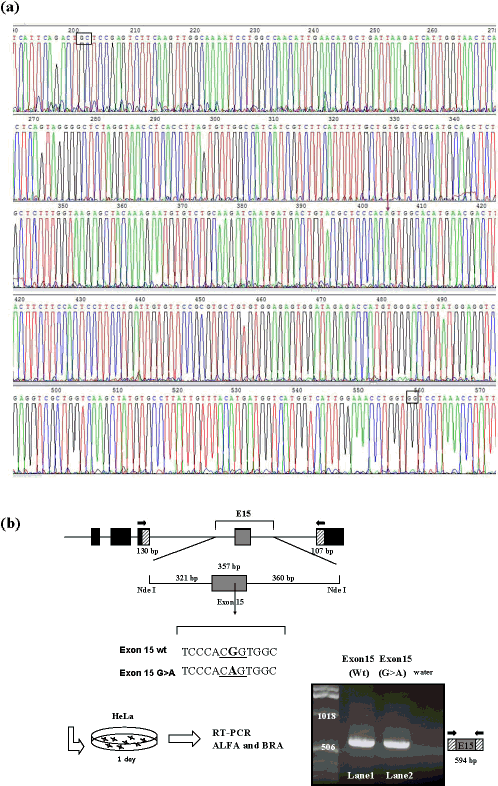

Supplement: Supplementary file 4 [file humu0031-1670-SD4.gif]

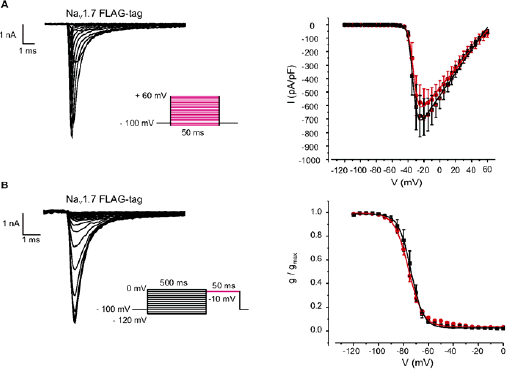

Supplement: Supplementary file 5 [file humu0031-1670-SD5.gif]

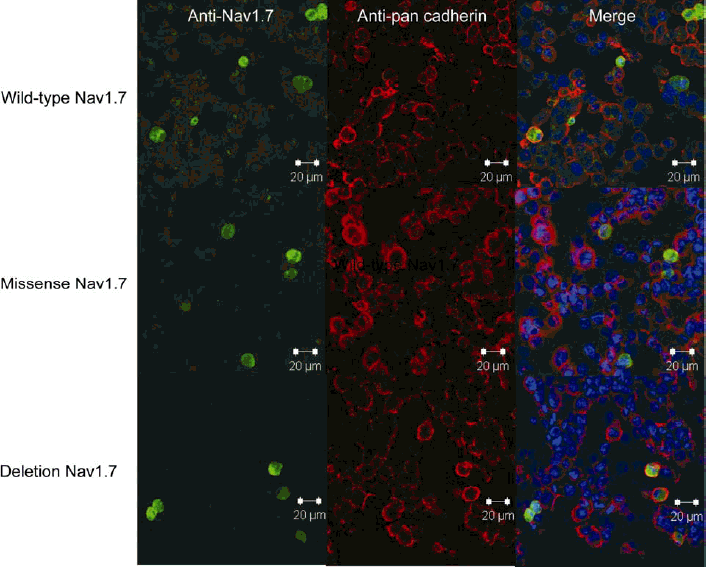

Supplement: Supplementary file 6 [file humu0031-1670-SD6.gif]
